# Cognitive ability and ideology join forces in the culture war: A model of opinion formation

**DOI:** 10.1093/pnasnexus/pgad205

**Published:** 2023-06-19

**Authors:** Kimmo Eriksson, Irina Vartanova, Isabela Hazin, Pontus Strimling

**Affiliations:** Institute for Futures Studies, Box 591, 101 31 Stockholm, Sweden; School of Education, Culture and Communication, Mälardalen University, Box 883, 721 23 Västerås, Sweden; Institute for Futures Studies, Box 591, 101 31 Stockholm, Sweden; Institute for Futures Studies, Box 591, 101 31 Stockholm, Sweden; Institute for Futures Studies, Box 591, 101 31 Stockholm, Sweden

**Keywords:** opinion formation, ideology, cognitive ability, public opinion, moral arguments

## Abstract

We propose a model of moral policy opinion formation that integrates both ideology and cognitive ability. The link from people's ideology to their opinions is assumed to go via a semantic processing of moral arguments that relies on the individual's cognitive ability. An implication of this model is that the relative quality of arguments that justify supporting vs. opposing a moral policy—the policy's “argument advantage”—is key to how opinions will be distributed in the population and develop over time. To test this implication, we combine polling data with measures of the argument advantage for 35 moral policies. Consistent with the opinion formation model, the argument advantage of a moral policy accounts for how public opinion moves over time, and how support for the policy ideologies varies across different ideological groups and levels of cognitive ability, including a strong interaction between ideology and cognitive ability.

**Significance Statement**

The relative prominence of right-wing moral opinions among people with lower cognitive ability is a well-studied yet poorly understood phenomenon. Here, we propose a dynamic model of opinion formation in which the processing of moral arguments has both ideological and cognitive components. Key in this model is which kinds of arguments justify a certain moral policy. We demonstrate the usefulness of the model by testing its predictions, at the population level, for 35 moral policies for which the justifying arguments are known. In particular, the interaction between ideological values and cognitive ability assumed by the model is observed in the data. This research offers an explanation to a long-standing puzzle in the intersection of psychology, political science, and sociology.

## Introduction

Should abortion be legal? Should books about homosexuality be banned from public libraries and schools? These are just two of a range of current issues on noneconomic political policy (hereafter: moral policy); other examples include the death penalty, euthanasia, gun rights, marijuana, sex education, pornography, etc. Strongly opposing opinions on these moral policy issues are typically seen as a divide between liberals and conservatives, sometimes referred to as the culture war (1). However, the connection between self-reported ideology and moral policy opinions is not very strong on the individual level (2). In other words, there are plenty of self-reported conservatives who support policies that are typically associated with liberals, and vice versa. Differences in opinions on moral policies between liberals and conservatives have been attributed to differences in underlying moral values, specifically, in how they rely on different moral foundations (3). But why are differences in opinions so small? Here we develop a model of opinion formation that, besides moral values, takes two other factors into account: the kind of moral arguments that justify each opinion and people's capacity to understand these arguments.

Our starting point is that opinions must be understood in a dynamical context, because people occasionally change their moral opinions. This is evidenced by studies that find opinion change over time even in a fixed cohort, as such change cannot be attributed to generational replacement (4). It is also evidenced by experiments showing that people may change their opinion on a moral issue after exposure to an argument for the opposing opinion (5, 6). These experiments further show that it matters what kind of argument it is. People are more likely to be swayed by arguments that are in line with the moral foundations that are associated with their ideology. Put differently, exposure to moral arguments gives people information about whether the values they hold would in fact justify another opinion on the issue at hand.

**Fig. 1.**
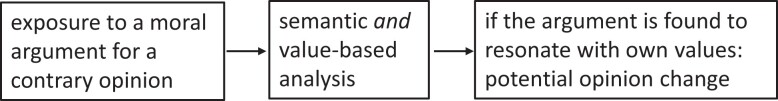
A model of opinion formation.

However, it is not a trivial task to properly understand an argument (7). We should therefore expect considerable individual variation in people's understanding of moral arguments. Indeed, a survey research has shown that people with lower scores on a cognitive (verbal) ability test are less clear about which arguments justify which opinions (8). We therefore propose a model of opinion formation based on exposure to moral arguments in which persuasion may occur only when the argument is of a kind that resonates with the individual's values and when the individual is able to understand what kind of argument it is and how it motivates a change of opinion. See Fig. 1.

At a general level, the basic features of this model align with prior work. For example, consider Zaller's classic receive-accept-sample (RAS) model of how people form opinions based on political messages (9). In the RAS model, the reception of political messages is assumed to be contingent on political awareness, a cognitive quality that helps individuals determine whether a message is consistent with their prior beliefs, which in turn affects their acceptance of the message. If we reinterpret political messages as exposure to arguments, prior beliefs as moral values, and political awareness as cognitive ability, we retrieve the model in Fig. 1. This reinterpretation is not only cosmetic, however. By focusing specifically on the moral policy domain, we can draw on how moral foundations theory distills the space of all moral arguments into a few basic kinds. We can measure the applicability of these few basic kinds of moral arguments for any given moral policy. Within the same framework, we can then study a different kind of question, namely, why public opinions vary *between different moral policies*.

Zaller's theory focuses on top-down political communication in the form of political messages sent by political elites and received by the general public, which may cause increasing differences in opinions between liberal and conservative members of the public (10). Top-down models beg the question how opinions among the elites are formed. By contrast, we assume that the same individual-level model applies to everyone, so that members of the elite may also change their opinions from exposure to arguments, and everyone who engages in a discussion is potentially both a sender and a receiver of arguments (11). Under these assumptions, the individual-level model generates population-level change through social dynamics that we have formally modeled elsewhere (12–14). This process of gradual opinion change may have been initiated by the modernization of moral values that followed upon the rise in prosperity after industrialization (13).

We shall describe how our model yields specific predictions about the change and distribution of opinions on specific moral policies in a specific population when given the data on: the *kinds of arguments* that are used to justify specific opinions; the population-level distribution of the *moral values* that connect with different kinds of arguments; and the distribution of the different levels of *cognitive ability* that determine individuals’ understanding of arguments they are exposed to.

### The kinds of arguments that are used to justify specific opinions

The moral foundations theory (15, 16) provides a conceptual framework for categorizing moral arguments into “individualizing” kinds (harm, fairness, and liberty) and “binding” kinds (authority, loyalty, and purity). They differ in how well they connect with most people's values. Specifically, individualizing kinds of arguments seem to be near-universally valued, whereas the binding kinds of arguments are generally less valued: many people hardly value them at all, and almost nobody values them more highly than the individualizing kinds of arguments (17, 18). Our assumption is that people are swayed only by arguments that they value. As every moral policy issue involves concerns about harm, fairness, or liberty, there will always be some relevant argument of the individualizing kind. Among those who care primarily about individualizing arguments, they will drive opinion change toward opinions that are supported by individualizing arguments. As that group interacts with the rest of the population, those who care about both kinds of arguments will increasingly be exposed specifically to arguments in favor of the opinions that are supported by individualizing arguments and therefore move toward those opinions. To understand the long-term change of opinions at the population level, it should therefore be sufficient to only consider which opinions are supported by individualizing kinds of arguments and ignore whatever arguments of the binding kinds that are also used for a given issue. In other words, a policy's justifiability by individualizing kinds of arguments should be a key determinant of its success at spreading in the population. This reasoning, including the assumption that binding arguments can be ignored, has been supported both by analysis of formal models (12) and empirical studies of opinion change (13, 18).

Our model focuses on within-generation change. However, assuming that young individuals obtain their starting opinions from the older generation's current opinions and that discussions and changes of opinion are more frequent among younger people than later in life, the process will also lead to between-generation changes in the same direction (13).

Note that there may be individualizing arguments both for and against a given moral policy. It is therefore necessary to quantify and compare how well each opinion on the policy is justified by individualizing arguments. We say that a moral policy has an *argument advantage* if individualizing arguments are more strongly for than against the policy. In the opposite case, we say that the moral policy has an argument disadvantage. In sum, our model predicts that moral policies will gain support over time if and only if they have an argument advantage (12). The argument advantage of any moral opinion can be measured using surveys, and such studies have found that measures are essentially rater-independent; for example, very similar argument advantage scores are obtained irrespective of whether the respondents are men or women, old or young, liberal or conservatives, or whether they live in the United States, Israel, or Brazil (8).

**Prediction 1 (Opinion trends on moral policies):** Public support increases for policies with an argument advantage and decreases for policies with an argument disadvantage.

Note that our model focuses on the role of moral arguments and disregards other factors behind opinion change, such as social movements (19), critical moments in a country's political history (20), and shifts in the media coverage of issues (21). While such factors undoubtedly cause temporary changes in opinions on moral policies, the assumption underlying Prediction 1 is that change in the long term is determined largely by a moral policy's argument advantage. Thus, our theory attempts to answer a question that is typically not addressed by other theories: “What is it about an opinion that makes it gain or lose in popularity in the long term?” Next we show that our theory also addresses another question with the same structure: “What is it about an opinion that makes it more common among liberals or among conservatives?”

### The population-level distribution of values that connect with different kinds of arguments

Above we mentioned that the binding kinds of moral arguments are much less valued than individualizing arguments in the population as a whole. However, moral foundations research has established that there are important individual differences that are closely associated with self-reported ideology on the liberal-conservative spectrum (17, 18). Specifically, there is a gradient from extreme liberals, who care very little about binding foundations but only about individualizing foundations, to extreme conservatives, who care almost as much about binding foundations as they care about individualizing foundations. According to our model, argument-driven opinion change should therefore be especially likely among liberals. It follows that liberals will be ahead of conservatives on the general opinion trend predicted above (13). In other words, whether a policy has an argument advantage or disadvantage should determine not only how opinions trend but also whether the strongest support for the policy is found among liberals or conservatives.

**Prediction 2 (Effect of ideology on opinions on moral policies):** Support for a policy with an argument advantage is stronger among liberals than conservatives; support for a policy with an argument disadvantage is weaker among liberals than conservatives.

### The distribution of different levels of cognitive ability

There is no consistent association between cognitive ability and self-reported ideology (22). Nevertheless, much empirical work has established that high cognitive ability, especially verbal ability, is associated with a higher probability of holding certain liberal moral opinions, such as tolerance of deviants (23–25). In other words, cognitive ability appears to complement moral values as a determinant of moral opinions. Our model provides an explanation as follows.

As discussed above, the more liberal people are, the more exclusively they rely on individualizing kinds of arguments. However, they can only know which arguments are individualizing if they understand the arguments. The implication is that opinions with an argument advantage will have a greater advantage among people with higher cognitive ability than among people with lower cognitive ability—and more so the more liberal people are. In other words, we should see a positive effect of cognitive ability on the probability of holding these opinions, and this effect should be stronger among liberals and weaker among conservatives. This amounts to a pair of predictions.

**Prediction 3 (Effect of cognitive ability on opinions on moral policies):** Support for a policy with an argument advantage is stronger among people with higher cognitive ability; support for a policy with an argument disadvantage is weaker among people with higher cognitive ability.

**Prediction 4 (Interaction of ideology and cognitive ability on opinions):** The cognitive ability gap favoring argument-advantaged opinions on moral policy decreases across the liberal-conservative spectrum.

Recall that our model also implies that policies with an argument advantage will be liberal policies (Prediction 2). Thus, Prediction 3 implies that it is specifically liberal policies that will have stronger support among people with higher cognitive ability. Hence, our model provides an explanation for the aforementioned positive association between cognitive ability and liberal moral opinions. There have been several previous attempts at explaining this association (26–28). However, our theory is unique in also predicting that the association is stronger among liberals. For example, consider the alternative hypothesis that arguments for liberal moral opinions are more cognitively demanding to understand than arguments for the conservative opinions on the same issues (27). While this hypothesis would explain that cognitive ability has an effect on moral opinions, it does not predict any interaction with ideology. Thus, whether Prediction 4 holds is a key test to distinguish our theory from alternatives.

Below we report an empirical study designed to test the predictions from our model in polling data from the General Social Survey (GSS). At the individual level, our theory concerns ideological affiliation (as a proxy for moral values) and cognitive ability. However, political opinions on specific opinions are also associated with many other individual variables that are included in the GSS, such as gender (29), age (30), race (31), education and income (32), media consumption (33), and interest in politics (34). We therefore include these variables as controls in the analysis.

## Results

**Fig. 2.**
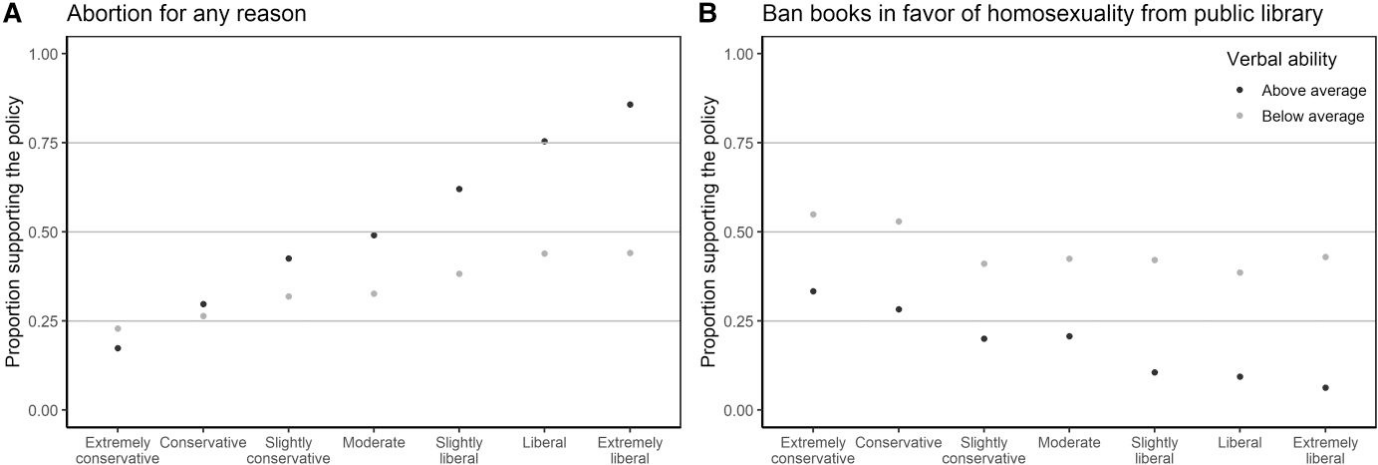
Support for two moral policies across ideological groups and levels of cognitive ability. Support for legal abortions is associated with higher cognitive ability, especially among liberals (**A**), while support for the removal of books is associated with lower cognitive ability, especially among liberals (**B**). Based on data pooled from all waves of the General Social Survey.

The predictions combine two units of analysis: individuals and moral policies. Individuals are assumed to vary in ideology and cognitive ability. The moral policies are assumed to vary in their argument advantage. Prior research has measured the argument advantage of many GSS items (8, 18), including the 35 GSS items on moral policy used in the present study (Table S1). Measures were obtained in surveys of how justifiable different opinions are by arguments based on harm, fairness, and liberty. For example, consider the policy that abortion be legal if the woman wants it. Arguments based on harm, fairness, and liberty were rated as more applicable to justify support of legal abortion than to justify opposition to legal abortion. Thus, legal abortion has an argument advantage. In line with Prediction 1, support for legal abortions increased from 35% in 1978 (the first year the item was included in the GSS) to 52% in 2018. In line with Predictions 2–4, support is higher among liberals, support is higher among people with verbal ability, and the verbal ability gap in support is especially large among liberals (Fig. 2A). An example of a moral policy with an argument disadvantage is to remove books about homosexuality from the library. Arguments based on harm, fairness, and liberty were rated as more applicable to justify opposition to removal of books about homosexuality than to justify support of this policy. In line with Prediction 1, support for removing books about homosexuality decreased from 40% in 1974 to 14% in 2018. In line with Predictions 2–4, support is lower among liberals, support is lower among people with verbal ability, and the verbal ability gap is especially large among liberals (Fig. 2B).

To test the predictions in greater generality, we analyze all 35 moral policies covered by the GSS. We conduct the analysis in two steps. In the first step, the units of analysis are individuals. For each policy, we perform a logistic regression analysis in which the probability that a respondent supports the policy is predicted by the year of the survey and the respondent's cognitive ability score and ideology (on the conservative-liberal spectrum) and their interaction. In the second step, the units of analysis are the policies themselves. We use correlational analyses to examine how the results from the first step may be explained by the argument advantage of each policy. In the analysis reported here, the first step controlled for the respondents education, income, media consumption, sex, race, and age. In the supplementary information (Fig. S1), we show that the main results are unchanged when we additionally control for interest in politics in a subsample of respondents for which this variable is available.

### The argument advantage of a policy determines the trend of its support in the population

**Fig. 3.**
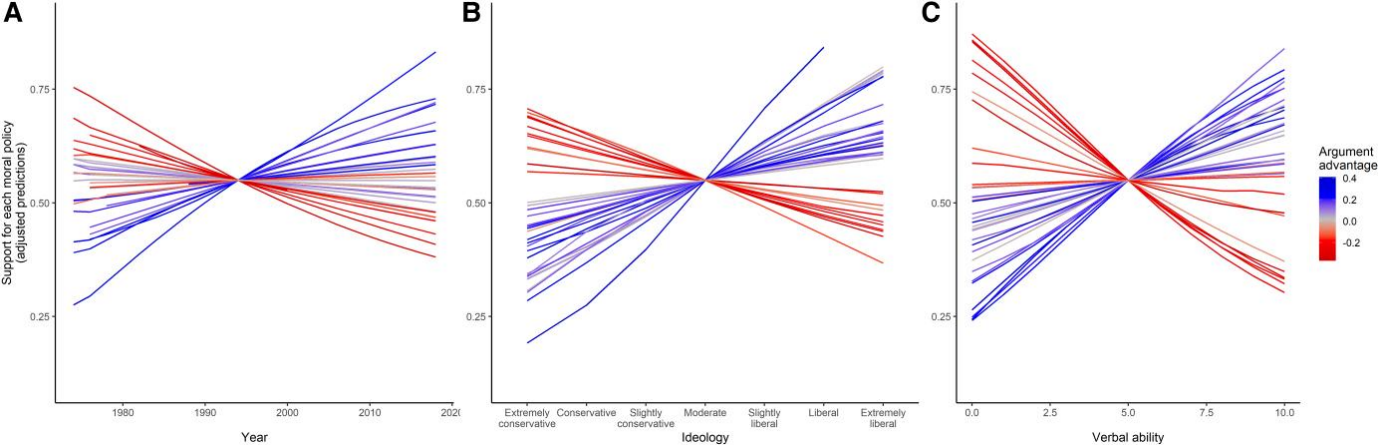
How the support for 35 moral policies varies with time, ideology, and cognitive ability. Lines show the estimated effects on the support for each moral policy of the year of the survey (**A**), of more liberal ideology (**B**), and of higher cognitive ability (**C**), controlling for gender, age, race, education, income, and media consumption. To highlight the differences in slopes, intercepts are adjusted so that all lines coincide in the same point. Lines referring to argument-advantaged policies tend to slope upward. Lines referring to argument-disadvantaged policies tend to slope downward.

Opinion trends are measured by the estimated effect of the year of the survey on support for a policy. In support of Prediction 1, opinion trends for various moral policies are positively correlated with the argument advantage of the policies, *r* = 0.79, 95% CI [0.62, 0.89], *n* = 35, *P* < 0.001. Specifically, argument-advantaged policies tend to become more popular over time, while argument-disadvantaged policies tend to become less popular over time (Fig. 3A).

### The argument advantage of a policy determines how support varies across ideological groups

In support of Prediction 2, the estimated effects of the respondents’ ideology on their support for various moral policies are positively correlated with the argument advantage of the policies, *r* = 0.80, 95% CI [0.64, 0.90], *n* = 35, *P* < 0.001. Specifically, argument-advantaged policies are more popular among liberals than conservatives, while argument-disadvantaged policies are more popular among conservatives than liberals (Fig. 3B).

### The argument advantage of a policy determines how support varies across levels of cognitive ability

In support of Prediction 3, the estimated effects of the respondents’ cognitive ability on their support for various moral policies are positively correlated with the argument advantage of the policies, *r* = 0.85, 95% CI [0.72, 0.92], *n* = 35, *P* < 0.001. Specifically, argument-advantaged policies are more popular among people with higher cognitive ability, while argument-disadvantaged policies are more popular among people with lower cognitive ability (Fig. 3C).

### Ideology and cognitive ability interact

**Fig. 4.**
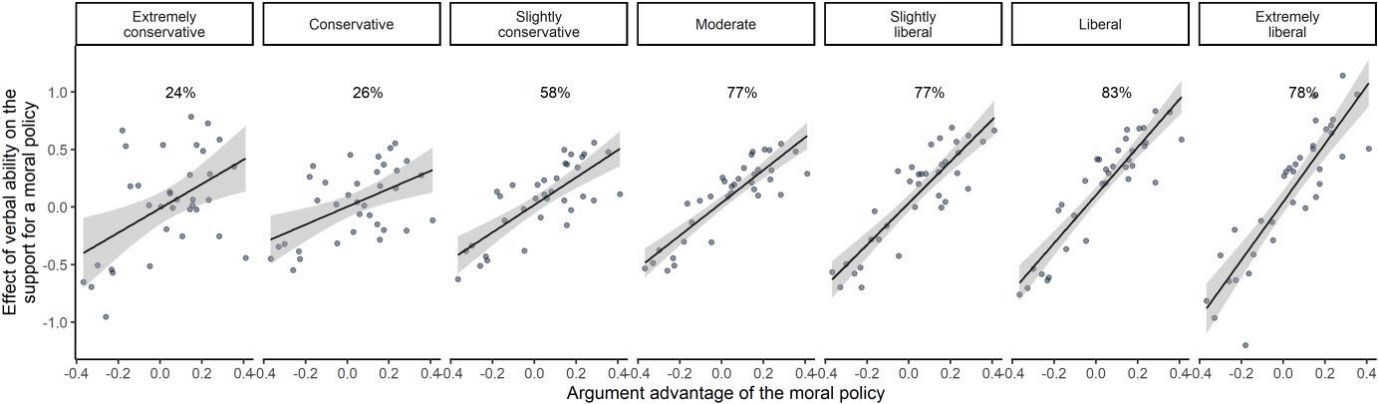
The effect of cognitive ability on the support of different moral policies in different ideological groups. Each panel represents an ideological group. Within an ideological group, each dot represents the estimated effect of cognitive ability (controlling for education, income, media consumption, sex, race, and age) on the support of a specific moral policy plotted against the policy's argument advantage. The regression lines indicate that higher cognitive ability is associated with stronger support for argument-advantaged policies and weaker support for argument-disadvantaged policies. The increase in slopes moving from the left panel to the right panel indicates that this systematic effect of cognitive ability is stronger in more liberal ideological groups. The percentages refer to *R*^2^, that is, the proportion of variance explained by the regression lines.

Prediction 4 concerns the interaction between ideology and cognitive ability in opinions about various moral policies. As expected, the estimated interactions are positively correlated with the argument advantages of the policies, *r* = 0.75, 95% CI [0.55, 0.86], *n* = 35, *P* < 0.001. To illustrate the interaction, Fig. 4 shows the effects of cognitive ability on the support for various moral policies estimated in each ideological group, plotted against the argument advantage of the policies. Note how the slope increases across the conservative-liberal spectrum to become very steep among extreme liberals.

## Discussion

Moral policies, such as policies about the legality and availability of abortions or books, may have a serious impact on people's lives (35, 36). Here we have argued that some policies are generally perceived to be advantageous with respect to their impact on harm, fairness, and liberty, and that these perceptions are key to explaining the distribution of opinions across individuals and the population-level change of opinions over time. Policies that are well justified by arguments referring to harm, fairness, and liberty gain in support over time, presumably because people care about these values. These policies are more popular among liberals than among conservatives, presumably because liberals care more strongly about these values than conservatives do. We have recently shown that the argument advantage measures can be turned into accurate predictions about the future (37), which provides additional support for the assumption that these measures capture how arguments are in fact used. Even when a new moral policy is proposed, it is possible that an argument analysis can yield an accurate prediction of whether the policy will be more popular among liberals or conservatives and whether it will gain in popularity over time. At present this method is limited to policies that are justified by moral arguments; it remains an open question how well it generalizes to, say, economic and environmental policies that may be justified by other kinds of arguments.

The main purpose of this paper was to use moral argument theory to gain a better understanding of the relation between cognitive ability and moral opinions. Our theory simultaneously provides an explanation for why people's cognitive ability impacts their moral policy opinions, on which policies the effect is largest, which opinions on these policies are favored by high cognitive ability, and whose opinions are most affected. Namely, cognitive ability helps people sort out how arguments for different positions fit their values. This has more impact on opinions on those policy issues where the most important values clearly fit better with one position than with the other, that is, where the policy has a clear argument advantage or disadvantage. It is the advantaged opinion that will be favored by high cognitive ability, and the effect is greatest among liberals. Our empirical findings are consistent with this theory. For a stark illustration of how the cognitive ability effect may vary across ideological groups, consider the results for legal abortion (Fig. 2B). Among conservatives, there is essentially no cognitive ability gap in the support for legal abortion. Among extreme liberals, by contrast, the cognitive ability gap is so large that the support for legal abortion among extreme liberals with low cognitive ability is less similar to other extreme liberals than to conservatives. This rules out the alternative explanations that attribute the effect of cognitive ability to a greater appeal of liberal opinions to smarter people. To be consistent with the data, any alternative explanation must be able to account for the remarkable interaction between cognitive ability and ideology. In the same vein, any alternative explanation must be able to account for the strong interaction with the argument advantages of the opinions.

The argument advantage of policies and the cognitive ability of the respondents were observed, not manipulated. Our theory provides a causal interpretation of the observed associations, but other causal interpretations are possible. To limit omitted variable bias, we controlled for several possible confounders of individuals’ cognitive ability, such as their education, income, and media consumption. The argument advantage of moral policies could potentially be confounded by, say, their total media coverage over the five decades covered in this study, but we are not aware of any available measures that are comparable across all moral policies.

Consistent with our assumption that the role of cognitive ability lies in the understanding of verbal arguments, prior research has found that political attitudes are better predicted by measures of verbal rather than nonverbal cognitive ability (23). The measure of cognitive ability that is available in the GSS is a brief vocabulary test called Wordsum; it is similar to the vocabulary subtest of the Wechsler Adult Intelligence Scale, which has a correlation above.80 with the overall test score (38). For this reason, Wordsum is very commonly used in studies that relate cognitive ability to political attitudes and political behavior (e.g. 22, 25, 39, 40). Nonetheless, it is a limitation that this measure does not explicitly measure comprehension of arguments. Note that the respondents with low scores on Wordsum may give less coherent responses to opinion questions (40). However, as this effect should be found both among liberals and conservatives, it cannot account for the key finding of an interaction between ideology and cognitive ability.

Another limitation of the GSS data is that self-reported ideology is only a proxy for how the individual values different kinds of moral arguments. A direct measure such as the Moral Foundations Questionnaire (17) would be more ideal.

## Materials and methods

### Experimental design

The objective of the study is to examine how well the argument advantage of a moral policy can account for how the support in the population for the policy changes over time and how it varies across ideological groups and different levels of cognitive ability in the population. To achieve this objective, we first identify a set of moral policies; second, we obtain measures of their argument advantage; third, we derive measures of how the support in the population for each policy changes over time and how it varies across ideological groups and different levels of cognitive ability in the population; and fourth, we examine how well the measures obtained in the second step predicts the measures obtained in the third step.

### Selection of GSS items

The selection of 35 GSS items was based on two criteria. First, the item must belong to a set of 98 items for which prior research has measured the argument advantage (see below). Second, the item must be about a moral policy for regulating people's behavior rather than about personal approval or disapproval of behavior. After exclusion of items of the latter kind, 35 items remained.

### Measures of the argument advantage of moral policies

Measures of the argument advantage for each of the 35 moral policies were taken from a prior study (18). These measures, with a theoretical range between −1 and 1, were in turn based on average responses to a prior survey (8). Every GSS item (e.g. “Homosexual couples should have the right to marry one another”) was judged by more than 100 participants. A list of arguments adapted from the Moral Foundations Questionnaire (17) was used; “Someone suffers emotionally” is an example of an argument of the harm kind, and “Someone is denied his or her rights” is an example of an argument of the fairness kind. For each opinion on the item, supporting vs. opposing a moral policy, the participants ticked all arguments that they believe apply to justify that opinion. For every kind of argument, the proportion of the sample that reported arguments of a given kind as applying to that opinion yielded a measure between 0 and 1 for the applicability of that kind of argument to that opinion. These measures are available at https://github.com/irinavrt/moral-args-appli. From these measures, the policy's advantage with respect to a given kind of arguments was calculated as their applicability to justify the policy minus their applicability to justify the opposite opinion. The total argument advantage was calculated as the average of the four advantages with respect to harm, violence, fairness, and liberty.

### Opinion data on moral policies

For the 35 selected items, we use all the data available from all waves of the GSS between the years 1974 and 2018 (41). The total number of respondents varied across items, ranging from 1,127 to 25,225. On items that include neutral responses (such as “neither agree nor disagree”) and/or graded responses (such as “slightly agree” and “strongly agree”), the data were dichotomized by omission of neutral responses and combination of graded responses, following prior research (13, 18).

### Individual factors

In the GSS, the respondents report their ideological affiliation on a seven-step scale from extremely conservative to extremely liberal, coded from 1 to 7 in the regression analyses so that the higher values mean more liberal. Following prior research (e.g. 22, 25, 39, 40), cognitive ability is measured by the GSS variable known as Wordsum. This is a score between 0 and 10, referring to the number of correct answers to the meaning of 10 English words (42). As control variables, we use education (number of years), family income (in constant dollars, log-transformed), news reading frequency (five-step scale from never to every day), sex (female or male), race (Black, White, or other), and age (in years). In a supplementary analysis, we also control for interest in politics (four-step scale from “not at all interested” to “very interested”). Note that the data on interest in politics was only available in some waves of the GSS, so the supplementary analysis is based on much less data.

### Statistical analysis

For each policy, we estimate the following logistic regression model in the full data set from all waves of the GSS: *logit(Opinion_i_)* = *β*_0_*+ β*_1_*Y* + *β*_2_*I_i_ + β*_3_*VA_i_ + β*_4_*I_i_VA_i_ + βX_i_*, where *Opinion_i_* is the opinion of individual *i*, *Y* is the year of the survey, *I_i_* is the ideology of individual *i*, *VA_i_* is the verbal ability of individual *i*, and *X_i_* represents the control variables. The ideology, verbal ability, education, age, family income, and news consumption variables were standardized with mean zero and unit standard deviation. The year variable was centered around the earliest survey year (i.e. 1974) and divided by 10. To obtain Fig. 4, we analyze the data from each of the seven ideological groups separately. Ideology then disappears as a variable in the logistic regression model, so that it becomes *logit(Opinion_i_)* = *β*_0_*+ β*_1_*Y + β*_2_*VA_i_ + βX_i_*.

In a second step, we calculate the correlations between effect estimates for each policy and the argument advantage of the policy. An alternative to this two-step analysis would have been to conduct a mixed-level analysis using the opinion data and argument advantage data on all policies simultaneously, including random effects on the policy level as well as on the respondent level. However, this model did not converge.

## Supplementary Material

pgad205_Supplementary_Data

## Data Availability

The GSS data is available at http://gss.norc.org. Argument advantage data is found in Table S1. All code to reproduce the results of this paper is available at https://github.com/IHazin/moral-opinions-cognitive-ability.
