# Supplementary material for: Cognitive ability and ideology join forces in the culture war: A model of opinion formation
Source: PNAS Nexus. 2023 Jun 19;2(6):pgad205. doi: 10.1093/pnasnexus/pgad205 (PMC10299893; doi:10.1093/pnasnexus/pgad205)
Supplement: pgad205_Supplementary_Data [file pgad205_supplementary_data.docx]

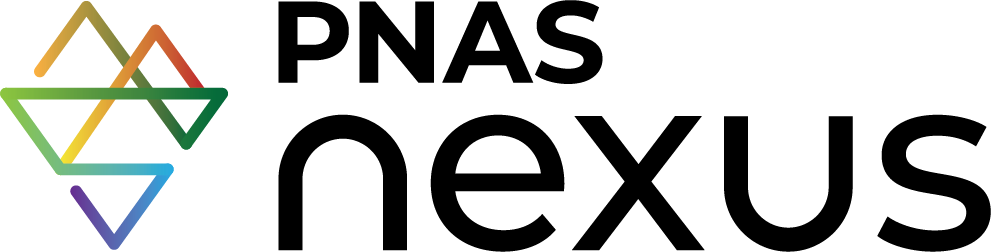


**Supporting Information for**

Cognitive ability and ideology join forces in the culture war: A model of opinion formation

Kimmo Eriksson, Irina Vartanova, Isabela Hazin, Pontus Strimling

Kimmo Eriksson

Email: kimmo.eriksson@iffs.se

**This PDF file includes:**

Table S1

Figure S1

Table S1. Argument advantage of the 35 GSS items on moral policy.

| **Moral policy code** | **Moral policy wording** | **Argument advantage** |
| --- | --- | --- |
| abany | Do you think it should be possible for a pregnant woman to obtain a legal abortion if the woman wants it for any reason? | 0.005 |
| abdefect | Do you think it should be possible for a pregnant woman to obtain a legal abortion if there is a strong chance of serious defect in the baby? | 0.030 |
| abhlth | Do you think it should be possible for a pregnant woman to obtain a legal abortion if the woman’s own health is seriously endangered by the pregnancy? | 0.156 |
| abnomore | Do you think it should be possible for a pregnant woman to obtain a legal abortion if she is married and does not want any more children? | 0.047 |
| abpoor | Do you think it should be possible for a pregnant woman to obtain a legal abortion if the family has a very low income and cannot afford any more children? | 0.060 |
| abrape | Do you think it should be possible for a pregnant woman to obtain a legal abortion if she became pregnant as a result of rape? | 0.143 |
| absingle | Do you think it should be possible for a pregnant woman to obtain a legal abortion if she is not married and does not want to marry the man? | 0.081 |
| cappun | Do you favor the death penalty for persons convicted of murder? | -0.143 |
| colath | There are always some people whose ideas are considered bad or dangerous by other people. For instance: somebody who is against all churches and religion – should such a person be allowed to teach in a college or university? | 0.238 |
| colcom | Consider a man who admits he is a Communist. Suppose he is teaching in a college. Should he be fired? | -0.300 |
| colhomo | Consider a man who admits that he is a homosexual. Should such a person be allowed to teach in a college or university? | 0.354 |
| colmil | Consider a person who advocates doing away with elections and letting the military run the country. Should such a person be allowed to teach in a college or university? | 0.157 |
| colmslm | Now consider a Muslim clergyman who preaches hatred of the United States. Should such a person be allowed to teach in a college or university? | 0.143 |
| colrac | Consider a person who believes that Blacks are genetically inferior. Should such a person be allowed to teach in a college or university? | 0.047 |
| forland | Foreigners should not be allowed to buy land in America. | -0.181 |
| grass | Do you think the use of marijuana should be made legal? | 0.232 |
| gunlaw | Would you favor a law which would require a person to obtain a police permit before he or she could buy a gun? | -0.052 |
| letdie1 | When a person has a disease that cannot be cured – do you think doctors should be allowed by law to end the patient’s life by some painless means if the patient and his family request it? | 0.175 |
| libath | There are always some people whose ideas are considered bad or dangerous by other people. For instance somebody who is against all churches and religion. If some people in your community suggested that a book he wrote against churches and religion should be taken out of your public library – would you favor removing this book? | -0.227 |
| libcom | Consider a man who admits he is a Communist. Suppose he wrote a book which is in your public library. Somebody in your community suggests that the book should be removed from the library. Would you favor removing it? | -0.260 |
| libhomo | Consider a man who admits that he is a homosexual. If some people in your community suggested that a book he wrote in favor of homosexuality should be taken out of your public library – would you favor removing this book? | -0.329 |
| libmil | Consider a person who advocates doing away with elections and letting the military run the country. Suppose he wrote a book advocating doing away with elections and letting the military run the country. Somebody in your community suggests that the book be removed from the public library. Would you favor removing it? | -0.232 |
| librac | Consider a person who believes that Blacks are genetically inferior. If some people in your community suggested that a book he wrote which said Blacks are inferior should be taken out of your public library – would you favor removing this book? | -0.048 |
| marhomo | Homosexual couples should have the right to marry one another. | 0.410 |
| pillok | Do you agree that methods of birth control should be available to teenagers between the ages of 14 and 16 if their parents do not approve? | 0.284 |
| pornlaw | Consider your feelings about pornography laws. Do you think it should be illegal? | -0.164 |
| racmar | Do you think there should be laws against marriages between African-Americans and whites? | -0.367 |
| sexeduc | Would you be for sex education in the public schools? | 0.106 |
| spkath | Consider somebody who is against all churches and religion. If such a person wanted to make a speech in your (city/town/community) against churches and religion – should he be allowed to speak? | 0.206 |
| spkcom | Consider a man who admits he is a Communist. Suppose this admitted Communist wanted to make a speech in your community. Should he be allowed to speak? | 0.229 |
| spkhomo | Consider a man who admits that he is a homosexual? Suppose this admitted homosexual wanted to make a speech in your community. Should he be allowed to speak? | 0.285 |
| spkmil | Consider a person who advocates doing away with elections and letting the military run the country. If such a person wanted to make a speech in your community – should he be allowed to speak? | 0.175 |
| spkmslm | Now consider a Muslim clergyman who preaches hatred of the United States. If such a person wanted to make a speech in your community preaching hatred of the United States - should he be allowed to speak? | 0.149 |
| spkrac | Consider a person who believes that Blacks are genetically inferior. If such a person wanted to make a speech in your community claiming that Blacks are inferior – should he be allowed to speak? | 0.014 |
| wirtap | Everything considered: would you say that – in general – you approve of wiretapping? | -0.105 |


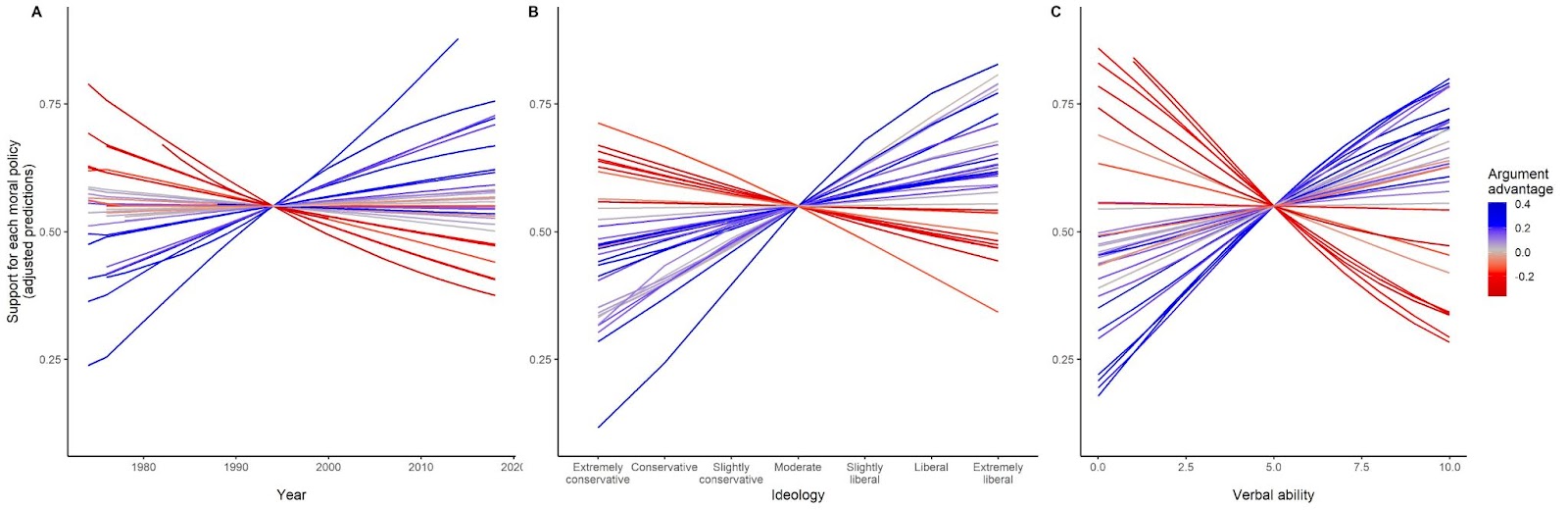


Figure S1. How the support for 34 moral policies varies with time, ideology, and cognitive ability. Lines show the estimated linear effects on the support for each moral policy of the year of the survey (A), of more liberal ideology (B), and of higher cognitive ability (C), controlling for gender, age, race, education, income, media consumption, *and interest in politics*. To highlight the differences in slopes, intercepts are adjusted so that all lines coincide in the same point. Blue lines refer to argument advantaged policies and tend to slope upward. Red lines refer to argument disadvantaged policies and tend to slope downward. (One policy, “forland” in Table S1, is excluded due to insufficient data.)
